# Mental health literacy and happiness among university students: a social work perspective to promoting well-being

**DOI:** 10.3389/fpsyt.2025.1541316

**Published:** 2025-03-26

**Authors:** Nurten Elkin, Ashifa Kariveliparambil Mohammed, Şenay Kılınçel, Ayse Mücella Soydan, Sultan Çakmak Tanrıver, Şebnem Çelik, Maharishi Ranganathan

**Affiliations:** ^1^ Child Development Department, Faculty of Health Sciences, Istanbul Gelisim University, Istanbul, Türkiye; ^2^ Department of Social Work, Faculty of Health Sciences, Istanbul Gelisim University, Istanbul, Türkiye; ^3^ Sakarya Child and Adolescent Psychiatry Institute, Istanbul, Sakarya, Türkiye; ^4^ Nursing Department, Faculty of Health Sciences, Istanbul Gelisim University, Istanbul, Türkiye; ^5^ Department of Nursing, Faculty of Health Sciences, Istanbul Gelisim University, Istanbul, Türkiye; ^6^ Department of Psychology, Christ University, Bangalore, India

**Keywords:** mental health literacy, happiness, university students, social work interventions, well-being

## Abstract

The present study tried to assess university students’ mental health literacy (MHL) and happiness levels and whether a relationship existed between these. The study used a descriptive quantitative methodology, utilizing Likert-type scales to collect data. A private university in Istanbul’s Faculty of Health Sciences had a sample of 443 students. Information was collected using a Personal Data Collection PR Form, the Oxford Happiness Questionnaire Short Form (OHQ-SF), and the Mental Health Literacy Scale (MHLS). Descriptive statistics and one-way analysis of variance (ANOVA) were used to analyze the data. The participants’ mean MHLS score was 23.00 ± 4.70, and the OHQ-SF score was 23.50 ± 4.70. We detected a significant difference in the MHL subscale owing to age, gender, department, class, maternal education, maternal employment status, income level, academic success, family attitude, smoking status, and exercise status. There were also differences in OHQ-SF scores by students’ department, class level, mother’s education level, father’s income level, academic success status, resident status, family attitude type smoking status, health perception of chronic illness, family history of chronic illness, exercise habit, nutritional status psychological problems, and family mental illness history. Knowledge-oriented and belief-oriented MHL subscales were weak but significantly negatively related, according to the findings. A weak correlation but a significant one was found for subscale Resource-Oriented MHL with happiness level and MHL Total. According to the above-stated research, people who can access mental health resources are more likely to be happy. These findings highlight how making mental health resources available could improve people’s mental well-being with a prolonged social work perspective. As happiness is a primary goal of life, more research contributing to our understanding of it is essential. The mental health literacy indicators for university students relate to realizing happiness and fostering well-being.

## 1 Introduction

The transition to university represents a critical developmental milestone for young adults, bringing significant academic, social, and psychological changes (1). This stage requires students to gain independence, handle academic workloads, and adapt to new social and economic settings. Many people are experiencing increased stress, anxiety, and other mental health problems (2). Young people aged 15–29 years are particularly at risk of mental health disorders. Notably, suicide is the second leading cause of death in this age group, as per WHO (3). Studies show that anxiety and depression are very common within university populations, with estimates suggesting that about 39% of students have at least one diagnosed mental illness (4). The widespread occurrence of this urges action and study among this population.

Mental health literacy (MHL) is one’s knowledge, skills, and attitude to identify, assess, and manage mental health issues, which is important for psychological well-being. It helps people notice signs, reduce stigma, and ask for help when necessary (5, 6). MHL is under-researched, especially in a university context, and there is no study yet on its relationship with other well-being, like happiness. Happiness is defined as the state of positive emotions and satisfaction with life sustained over time, which is key to mental health (7). Research has shown that being happy can help beach those bad times and boost well-being, which, in turn, improves academic performance, social life, etc. Very few studies have explored the relationship between MHL and the happiness of university students (8), which is an important gap in the literature.

This research is important for both individual and societal welfare. Improving MHL among students can help them manage their mental health better, seek help on time, and adopt healthier coping strategies. By helping individuals understand mental health better, MHL can reduce the stigma associated with mental illness that threatens help-seeking in several cultures and societies (9). At the level of society, universities are seen as a miniature version of society, enhanced by the possibility of testing interventions that can subsequently be scaled up (10). Having a high level of mental health literacy (MHL) enables students to not only do better academically but also help them deal with mental health-related problems throughout life and, thus, build healthier communities.

In addition, happiness is increasingly seen as a determinant of mental health and a predictor of success in many spheres of life. At a university, happier students are likelier to do well in school, have friends, and participate on campus (11). Being aware of things that can lead to one’s happiness, particularly concerning mental health literacy, can lead to doing something useful for the academic setup. Happiness may be thought of as an end goal. Nevertheless, it may also be considered a means target as it builds resilience and buffers stress (12).

Even though MHL and happiness are clearly important, not much research exists to examine the link between the two. This gap hinders our ability to accurately assess how knowledge, attitudes, and access to mental health resources are translated to emotional or psychological well-being (13). Looking into this connection is relevant to university students who are at a developmental stage of identity formation and exploring life goals while becoming subject to increased exposure to stressors (14).

The link between MHL and happiness is academically meaningful and practically beneficial. Universities are key to influencing mental health outcomes across generations (2). If these issues are not attended to, the students will suffer from academic burnout and unsatisfactory interpersonal relationships, together with mental health conditions in the future. Through investigating the link between MHL and happiness, the study intends to facilitate a targeted intervention that university policies and programs can adopt. This is especially useful because the evidence suggests that education and intervention can improve mental health and quality of life.

This study is also necessitated by the difficulties involved in conceptualizing happiness. Happiness is more subjective, unlike physical health, which is assessed through objective measures. Happiness depends upon a person’s personality, social relations, and culture. By looking at how MHL interacts with these factors, we can better understand happiness. Additionally, the results of this research may pave the way for effective and relevant strategies to promote mental health in a culturally sensitive manner.

### 1.1 Objectives of the study

1. To evaluate the levels of mental health literacy among university students and identify gaps in their knowledge, attitudes, and behaviors related to mental health.
2. Measuring happiness levels by university students is an inquiry to assess emotional and psychological indicators of flowering mental health.
3. To investigate the relationship between mental health literacy and happiness, analyzing how mental health knowledge and practices influence students’ well-being.
4. To examine the demographic, socioeconomic, and behavioral factors that shape mental health literacy and happiness among university students.
5. To propose evidence-based recommendations for developing interventions and policies that improve mental health literacy and enhance happiness among university students.

## 2 Review of literature

MHL is an emerging research area examining the connection between happiness and mental health literacy. University students, in particular, are uniquely vulnerable to mental health issues. This assessment analyzes the literature on MHL, its dimensions, and happiness, including the significance of context and demographic factors (15).

Mental health literacy refers to knowing about the mental illness, implying being able to identify a particular mental illness, know about the available treatment options, and attitude facilitating help (16). The term ‘stress’ refers to a multidimensional construct important for mental health management (17). In universities, students suffer from various stressors such as academics, social adaptation, and finances. Research shows that low mental health literacy is linked to poorer mental health among students (18). Many university students are unable to identify symptoms of the common disorders of anxiety and depression, which delays their help-seeking behavior. Moreover, like the other findings, stigmas and misunderstandings regarding mental health continue to limit access to care (19). According to Wei and his colleagues’ research paper published in 2015, higher Mental Health Literacy is associated with improved utilization of counseling services (20).

Although it is important, MHL is not sufficiently covered in many schools. Programs that promote mental health literacy, psychoeducation, peer counselling, and stigma reduction have shown promising results but are not universally implemented (21). For instance, students exposed to mental health literacy interventions are more likely to seek help and have less stigma toward mental illness (22).

Happiness is often understood to be an enduring experience of positive emotions and life satisfaction. It is a key element of mental health. It includes one’s feelings and evaluation of life (5). In the context of university life, happiness has been related to academic success, resilience, and social engagement, and a student at a university feels less happy due to stressors like workload, peer competition, and financial instability (23). Happiness does not only depend on luck. It depends on psychological and behavioral causes, too. According to Jiang et al. (24), students with a strong support system, regular exercise, and life purpose felt happier than average. This indicates that happiness is a variable that can be altered due to internal and external factors or influences.

The present research suggests that mental health might be important for happiness. Someone with a higher MHL (Mental Health Literacy) can better manage stress symptoms and experience positive moods (25). Moreover, information on mental health encourages adaptive coping strategies like seeking support or engaging in self-care which is linked with greater life satisfaction and emotional well-being.

Reavley and Jorm (26) propose that mental health literacy contributes to happiness in an indirect manner. The way they do so is by reducing the stigma attached to mental health problems, which consequently facilitates the use of mental health resources. When people understand mental health conditions and feel capable of dealing with them, they can maintain positive mental health. On the flip side, having low MHL can lead to misconceptions, avoidance, and delays in addressing the issues, consequently heightening distress and lowering happiness. Research into this area may be limited, but the result highlights a possible link between MHL and happiness. The explanation for this relationship is still unclear in a university context. For instance, does MHL lead to decreased depression, or does it cause increased well-being, such as confidence, social support, and resilience, for the individual?

Many demographic and behavioral variables affect both MHL and happiness. The most important determinants of mental health include age, sex, and wealth. According to Gagnon et al. (27), females may have higher mental health literacy than males, but they also report experiencing higher stress and anxiety, which suggests that knowledge is not everything. Socioeconomic factors are associated with financial constraints, access to mental health-related resources, and overall life satisfaction, which forms the basis of the relationship between MHL and happiness (28). Physical activity, connections with others, and doing meaningful things also matter a lot. Regular exercise can improve MHL (mental health literacy) and happiness by reducing anxiety (29). Social support networks help enhance mental health literacy by mutual sharing of knowledge and information while supporting happiness through emotional and instrumental support.

Another important consideration is cultural context. In societies where collective idealism is more celebrated, the stigma against mental illness may be more pronounced (30). MHL intervention here needs to address barriers to help-seeking by creating more help-giving. Culturally tailored mental health literacy (MHL) programs are more effective than universal programs at reducing stigma and promoting positive mental health outcomes (31). While previous studies provide a better understanding of MHL and happiness, there are still gaps. The author writes that research exploring the direct relationship between mental health literacy and happiness is limited among university students. Also, the role of mediating factors like self-efficacy or resilience is unclear. There is a need for longitudinal studies to determine the causality and the long-term effects of MHL interventions on happiness and mental health. Also, there is a lack of studies investigating how institutional factors, like university policies and mental campus health services, influence MHL and happiness. These contextual factors underpinning the issue will help formulate the solutions.

It is evident from the literature that mental health literacy plays an essential role in mental health and happiness. Individuals can tackle their mental health issues and become more robust thanks to High MHL rising to the occasion, ultimately strengthening their lasting happiness. How MHL associates with happiness is not studied much especially in students. The study aimed to shed light on the relationship between mental health literacy and happiness in a university environment while considering demographic and behavioral variables. By participating in this evolving research area, the examination seeks to speak about evidence-based procedures to promote pupil well-being and establish positive mental health outcomes.

Based on the Review of literature the following research questions were developed:

How does mental health literacy (MHL) influence the happiness levels of university students, and do these effects vary based on age and gender?

What is the relationship between student employment status and happiness levels, and how does MHL moderate this?

To what extent do family attitudes toward mental health impact university students’ mental health literacy and overall happiness?

How do different lifestyle choices correlate with mental health literacy and happiness among university students?

How does academic success shape university students’ mental health, literacy, and happiness?

## 3 Methods and materials

### 3.1 Research Design

This study, which aims to evaluate university students’ mental health literacy and happiness levels, is a descriptive study conducted with a quantitative design and Likert-type scales. Data were collected through face-to-face survey methods. An attempt was made to reach the entire population, but due to reasons such as unwillingness to continue, incomplete responses, and absenteeism, 752 students were excluded from the study. The sample size was calculated for a known population with a 95% confidence interval and a 5% margin of error. As a result of these calculations, the minimum sample size was determined to be 291 people based on the total number of individuals in the population. Students who left the survey incomplete or did not wish to continue were excluded from the study, and the data were collected based on voluntary participation. Missing data resulting from incomplete markings in the face-to-face surveys are noted in the tables.

### 3.2 Study population and sample

Study Population and Sample. The study population in this research consists of private university students who enrolled in Istanbul during the spring semester of the 2023–2024 academic year. A stratified random sampling of main subgroups will select the final sample of 443 students. Researchers divided students on the basis of demographics and academic characteristics, namely age, gender, year of study, and academic program, to ensure diversity and representation in the student population. The sampling method ensured that students from different years (freshman, sophomore, junior, senior) and varied fields of the university were adequately represented. This made the findings more applicable to undergraduate students at private universities in Istanbul.

### 3.3 Data collection instruments

In this study, a Personal Information Form, the Short Form of the Oxford Happiness Questionnaire (OHQ-SF) (32, 32), and the Mental Health Literacy Scale (MHLS) (33) were used.

Personal Information Form: The 30-item Personal Information Form developed by the researchers includes questions about the participants’ demographic characteristics, smoking habits, alcohol use, chronic illnesses of the participant or their family, exercise habits, healthy eating habits, and psychological health of the participant or their family.

Oxford Happiness Questionnaire Short Form (OHQ-SF): The Oxford Happiness Questionnaire Short Form, developed by Hills and Argyle (32), is an 8-item scale assessing happiness. This scale was translated into Turkish and adapted by Doğan and Çötok (34). As a result of item analysis, item 4 (“I do not think I look attractive”) was removed due to a total correlation of.17, and the scale was analyzed with 7 items. The item-total correlation values of the scale ranged between.36 and.55 (34). The scale uses a 5-point Likert-type rating system: (1- Strongly Disagree, 2- Disagree, 3- Slightly Agree, 4- Agree, 5- Strongly Agree). Items 1 and 7 are reverse-coded, and a high score indicates higher happiness. The internal consistency coefficient of the scale was calculated as 74 using Cronbach’s alpha, indicating sufficient reliability. The test-retest reliability coefficient was also found to be.85, demonstrating the scale’s consistency over time.

### 3.4 Mental Health Literacy Scale (MHLS)

The Mental Health Literacy Scale (MHLS) developed by Jung et al. (33) consists of 26 items and three subscales. During the Turkish validation and reliability study conducted by Göktaş et al. (35), two items (items 9 and 14) were removed following Exploratory Factor Analysis, and two more items (items 11 and 18) were excluded after Confirmatory Factor Analysis (35). The internal consistency coefficient (Cronbach’s alpha) of the scale was found to be.71. The scale consists of 22 items divided into three subscales: Knowledge-Oriented MHLS (11 items), Belief-Oriented MHLS (8 items), and Resource-Oriented MHLS (4 items). The Belief-Oriented subscale is reverse-coded. The first 18 questions, comprising the first and second subscales, are rated on a Likert scale: “1- Strongly Agree,” “2- Agree,” “3- Neutral,” “4- Disagree,” “5- Strongly Disagree,” “6- Do not Know.” The last four questions of the Resource-Oriented subscale are answered with “yes” or “no.”.

### 3.5 Data analysis

This study analyzed data using descriptive statistics and one-way ANOVA. The ANOVA test was used to determine whether there were statistically significant differences between groups, and the F and p values were evaluated. Variables such as gender, education level, chronic illness, smoking, healthy eating, and medication use were examined. The results indicated a significant relationship between healthy eating, chronic illness, and happiness levels (measured by OHQ-SF), while no significant differences were found for other variables. The analyses are presented in tables.

## 4 Result

This study’s analysis section will cover the nature of the relationship between mental health literacy (MHL) and the happiness levels of university students. Analysis of selected socio-demographics, lifestyle behavior, and psychological characteristics assesses their effects on the Mental Health Literacy Scale (MHLS) and Oxford Happiness Questionnaire (OHQ-SF) scores. Descriptive statistics, ANOVA, and correlation statistical techniques will be used to determine the significance of the result. The study results provide a better understanding of mental health awareness, resource utilization, belief systems, and emotional states among students.

The socio-demographic characteristics of the study participants are depicted in **Table 1**. More than half of the respondents aged between 21–25 belong to the 61.5%. Only a few respondents were aged between 26–30 (5.6%) and above 30 (1.3%). The Sample had more women (60.9%) than men (39.1%). The majority of participants were single (94.6%); a smaller portion of them were married (5.0%); and the least was widowed/divorced (0.5%). Most students (74.0%) claimed not to be employed, while 26.0% were employed. Among the surveyed population, 75.2% indicated having an average income, while 20.8% reported a good income and 4.1% high income. Academic performance was rated mostly average (52.8%) and sound (46.5%), while only 0.7% rated it as poor. Most of the individuals lived with their family (62.3%), whereas (24.2%) lived in a dormitory, (4.5%) lived with friends, and (9.0%) lived in other accommodations; most of the participants lived in a city (69.8%), district (25.5%), and village (4.7%). Most families described their attitudes as protective (38.8%) and democratic (30.0%). Less often, they reported their family attitude as authoritarian (23.5%) and inconsistent (7.7%). The most prevalent type of family arrangement was the nuclear type, which constituted about 78.6% of the family structure. Traditional family systems were reported by 15.6% of the respondents. While 2.7% were from a single parent and 3.2% were from other family arrangements, this descriptive profile provides valuable information about the interaction between socio-demographic characteristics with mental health literacy and happiness among the participants.

**Table 1:** Socio-demographic profile.

	Variable Responses	Frequency	Percentage
Age	17-20	139	31.3
	21-25	272	61.5
	26-30	25	5.6
	Above 30	6	1.3
Gender	Woman	270	60.9
	Man	173	39.1
Marital Status	Married	22	5.0
	Single	419	94.6
	Widowed-Divorced	2	0.5
Employment Status	Yes	115	26.0
	No	328	74.0
	Good	92	20.8
Income Level	Average	333	75.2
	High	18	4.1
	Good	206	46.5
Academic Success	Average	234	52.8
	Poor	3	0.7
	Family	276	62.3
Living Situation	Dormitory	107	24.2
	With a Friend	20	4.5
	Other	40	9.0
Place of Residence	Village	21	4.7
	District	113	25.5
	City	309	69.8
Family Attitude	Authoritarian	104	23.5
	Democratic	133	30.0
	Inconsistent	34	7.7
	Protective	172	38.8
Family Structure	Nuclear	348	78.6
	Traditional	69	15.6
	Single Parent	12	2.7

The participants’ lifestyle, health, and psychological characteristics give great insights (**Table 2**). Most of the participants (74.7%) reported that they did not smoke, while (25.3%) reported smoking. Of the participants, 75.6% practiced non-drinking, while 24.4% reported drinking alcohol. Based on these findings show that most people in the population do not smoke or drink. However, quite a lot of the participants do smoke and drink. Over 61.4% of respondents claimed they perceived their health to be good. Of the total respondents, 39 percent considered their health above average, while 368 percent rated theirs as average. Only a tiny fraction, 1.8 percent, rated theirs as bad. Most participants appear to have a positive perception of their health, which may contribute to their happiness and mental well-being. 11.8% of the participants reported chronic illness, while the majority (88.2%) reported no chronic illness. The participants’ perception of their health status was positive in this situation, consistent with the low prevalence of chronic conditions. In relation to chronic disease family history, the response was fairly balanced, as 48.1% had a family history of chronic disease, whereas 51.9% did not have a family history of chronic illnesses. Out of the total sample, 11.1% used medication, while 88.9% did not. This is consistent with the overall trend towards good health and low chronic illness. However, a segment of our population that relies on medication may suffer from a health condition that negatively affects their mental health, literacy, or happiness. According to our findings, 22.8% of participants exercised regularly, while a considerable % of the population, 77.2%, did not exercise regularly. Limited levels of physical activity participation are a potential concern as participation in physical activity is associated with a beneficial increase in mental health. Regarding healthy eating habits, 53.0% of participants followed a healthy diet, while 47.0% did not eat healthily. Just over half the population adhered to healthy eating, which leaves a large percentage of the population who are likely able to eat more healthily. Almost one-fourth of the study’s participants were found to have had psychological problems, according to 24.4% of the participants. On the other hand, 75.6% of people face no psychological issues. Support status was especially low, with just 18.7% of the respondents stating that they received support, while 81.3% stated there was no support. The participants reveal a lack of support systems. Consequently, it could adversely affect their offer when dealing with psychological or emotional issues. Finally, 11.1% of participants said their family has a mental health problem, while 88.9% said their family does not have any. The indication is that most of the respondents do not have exposure to mental health problems in their family. However the number might be less for some. This extensive analysis of lifestyle, health, and psychological characteristics is a great protocol for understanding the relationship between mental health literacy and happiness levels in university students.

**Table 2:** Lifestyle, health, and psychological characteristics of the participants.

Variables	Responses	Frequency	Percentage
Smoking	Yes	112	25.3
	No	330	74.7
Alcohol Use	Yes	108	24.4
	No	335	75.6
Perceived Health Status	Good	272	61.4
	Average	163	36.8
	Bad	8	1.8
Chronic Illness	Yes	52	11.8
	No	390	88.2
Family History of Chronic Illness	Yes	213	48.1
	No	230	51.9
Medication Use	Yes	49	11.1
	No	394	88.9
Exercise	Yes	101	22.8
	No	342	77.2
Healthy Eating	Yes	235	53.0
	No	208	47.0
Psychological Problems	Yes	108	24.4
	No	335	75.6
Support Status	Yes	28	18.7
	No	122	81.3
Family Mental Health Problems	Yes	49	11.1
	No	394	88.9

The comparison of mental health literacy (MHL) subscale scores and happiness levels as measured by OHQ-SF across age groups is shown in **Table 3**. One MHL subscale has a significant difference, whereas other subscales and happiness scores do not differ significantly. A statistical analysis showed that the Knowledge-Oriented MHLS scores varied significantly according to age (F = 11.317, p < 0.001). Those aged 21–25 had the highest mean score of 8.74 and an SD of 1.69. This indicates they were more aware of mental health knowledge. The age bracket 17–20 and the age group 26–30 scored lower, with M = 7.73, SD = 1.774, and M = 7.75, SD = 1.962, respectively. Participants aged 30 and above scored slightly higher (M = 8.57, SD = 1.813), though the sample size for this group was very small (N = 7). No difference was found across age brackets in the Belief-Oriented MHLS scores (F = 1.476, p = 0.22). The difference between the means of these groups was 0.48, with positive effect size being only meaningful in the 26-30 group because the higher mean was not in that 30 and above group. The 21-25 year-old had a mean score of 6.14 (SD = 1.937), while the 17-20 year-old had a mean score of 6.42 (SD = 1.281).

**Table 3:** Comparison of MHLS and OHQ-SF scores by students’ age.

Variable	Age Group	N	Mean	(SD)	F	p
Knowledge-Oriented MHLS	17-20	138	7.73	1.774	11.317	< .001 *
	21-25	270	8.74	1.69		
	26-30	24	7.75	1.962		
	30 and above	7	8.57	1.813		
Belief-Oriented MHLS	17-20	137	6.42	1.281	1.476	0.22
	21-25	269	6.14	1.937		
	26-30	25	6.52	1.085		
	30 and above	7	7.0	1.0		
Resource-Oriented MHLS	17-20	139	2.3	0.882	0.798	0.496
	21-25	270	2.4	0.851		
	26-30	25	2.36	0.907		
	30 and above	7	2.71	0.488		
OHQ-SF	17-20	138	24.12	4.35	1.799	0.147
	21-25	272	23.13	4.913		
	26-30	25	23.96	4.247		
	30 and above	7	25.29	2.928		

*p *< .001 (*). The F-test for Knowledge-Oriented MHLS is statistically significant at *p *< .001.

The Resource-Oriented MHLS scores showed no significant differences across age groups (F = 0.798, p = 0.496). People of all age groups scored the same on the test. However, those aged 30 and above scored slightly higher (M = 2.71, SD = 0.488). The group of people who were 21-25 was found to have a mean score of 2.4 (SD=0.851), while the 17-20 and 26-30 groups happened to have similar mean scores of 2.3 (SD=0.882) and 2.36 (SD=0.907) respectively. No statistically significant differences in happiness (OHQ-SF scores) between age groups (F = 1.799, p = 0.147) exist. The mean score revealed similarities, where the maximum mean of 30 and above group (M = 25.29, SD = 2.928) followed by the 17–20 group (M = 24.12, SD = 4.35). The happiness score for participants between 21 and 25 was slightly lower (M = 23.13, SD = 4.913), and the score for participants aged 26 to 30 was 23.96 (SD = 4.247).

**Table 4** compares mental health literacy (MHL) subscales and happiness levels (OHQ-SF) of gender groups. There exists a significant gender difference in one of the MHL subscales, while others and the happiness levels of men and women were similar. Both genders significantly differed in Knowledge-Oriented MHLS score F=22.5908, p<0.001. Women had a significantly higher average score of 8.68 compared to men, whose average score was 7.86. Women have more awareness and knowledge of mental health than men. The Belief-Oriented MHLS scores did not differ much between genders (F = 0.0249, p = 0.875). Women scored 6.25 (SD = 1.733) while men scored 6.28 (SD = 1.675), meaning the attitudes and beliefs of both women and men about mental illness and its plight were similar. There was no significant difference in the Resource-Oriented MHLS scores between males and females (F = 1.2983, p = 0.255). The difference was statistically insignificant, while men scored somewhat higher (M=2.43, SD=0.79) than women (M=2.34, SD=0.901). This shows that both men and women are equally familiar with mental health services. Happiness levels, as given OHQ-SF, did not differ significantly between the genders (F = 0.3023, p = 0.583). The mean happiness score for women was 23.62 (SD 4.932), which is not much different from that of men at 23.37 (SD 4.316). According to this finding, both males and females are equally happy.

**Table 4:** Comparison of MHLS and OHQ-SF scores by gender.

Variable	Gender	N	Mean (M)	SD	F	p
Knowledge-Oriented MHLS	Woman	269	8.68	1.558	22.5908	< .001
	Man	170	7.86	2.02		
Belief-Oriented MHLS	Woman	268	6.25	1.733	0.0249	0.875
	Man	170	6.28	1.675		
Resource-Oriented MHLS	Woman	270	2.34	0.901	1.2983	0.255
	Man	171	2.43	0.79		
OHQ-SF	Woman	270	23.62	4.932	0.3023	0.583
	Man	172	23.37	4.316		

**Table 5** displays MHL subscales and happiness (OHQ-SF) scores obtained by students according to their employment status. The Knowledge-Oriented MHLS values of employed and unemployed students did not significantly differ (F = 2.225, p = 0.137). The average score of employed students was 8.15 (SD = 1.924), less than that of unemployed students, 8.44 (SD = 1.743). However, a statistically significant difference was found in the Belief-Oriented MHLS subscale (F = 7.15, p = 0.008). Students with no jobs scored more (M = 6.39, SD = 1.596) than those with jobs (M = 5.89, SD = 1.957). The employed students (M = 2.4, SD = 0.896) did not score differently from unemployed students (M = 2.37, SD = 0.848; F = 0.14, p = 0.708) on the Resource-Oriented MHLS subscale. Therefore, employed and unemployed students have the same familiarity with mental health resources. Like that, there were no sizeable differences in happiness scores (OHQ-SF) across employment status (F=0.11, p=0.741). The average happiness score of employed students was 23.39 (SD = 4.794), which was not too different from that of unemployed students (M = 23.56, SD = 4.672). So, based on the findings, it can be said that although beliefs regarding mental health differ significantly on the basis of employment status, knowledge-oriented and resource-oriented mental health literacy, and happiness do not differ significantly.

**Table 5:** Comparison of MHLS and OHQ-SF scores by students’ employment status.

Variable	Employment Status	N	Mean (M)	SD	F	p
Knowledge-Oriented MHLS	Yes	114	8.15	1.924	2.225	0.137
	No	325	8.44	1.743		
Belief-Oriented MHLS	Yes	114	5.89	1.957	7.15	0.008
	No	324	6.39	1.596		
Resource-Oriented MHLS	Yes	115	2.4	0.896	0.14	0.708
	No	326	2.37	0.848		
OHQ-SF	Yes	114	23.39	4.794	0.11	0.741
	No	328	23.56	4.672		

**Table 6** displays MHL subscales and OHQ-SF scores according to students’ academic success. There were no significant differences in Knowledge-Oriented MHLS scores based on the level of academic success (F=1.544, p=0.215). Those with good academic success rated their academic success at 8.52 (SD = 1.706), whereas those with academic success rated their academic success at 8.23 (SD = 1.855). Lastly, those with poor academic success rated their academic success at 8.0 (SD = 2.646). Likewise, the Belief-Oriented MHLS (F = 0.282, p = 0.754) score does not differ significantly among the groups. The good academic success group score was M = 6.26 (SD = 1.716). The average group score was M = 6.25 (SD = 1.716). Finally, the poor academic success group score was M = 7.0 (SD = 0.0). However, the sample size for this group is very small. Concerning the Resource-Oriented subscale of MHLS, significant differences were obtained (p = 0.02, F = 3.953). Students with poor academic success were significantly lower (M = 1.0, SD = 1.0) compared to those with good (M = 2.37, SD = 0.84) and average academic success (M = 2.39, SD = 0.865). This means students with low academic performance probably know less about mental health resources.

**Table 6:** Comparison of MHLS and OHQ-SF scores by students’ academic success.

Variable	Academic Success	N	Mean (M)	SD	F	p
Knowledge-Oriented MHLS	Good	204	8.52	1.706	1.544	0.215
	Average	232	8.23	1.855		
	Bad	3	8.0	2.646		
Belief-Oriented MHLS	Good	203	6.26	1.716	0.282	0.754
	Average	232	6.25	1.716		
	Bad	3	7.0	0.0		
Resource-Oriented MHLS	Good	205	2.37	0.84	3.953	0.02
	Average	233	2.39	0.865		
	Bad	3	1.0	1.0		
OHQ-SF	Good	206	24.22	4.835	4.696	0.01
	Average	233	22.88	4.514		
	Bad	3	25.0	2.646		

The happiness scores (OHQ-SF) significantly differed due to the academic success levels (F = 4.696, p = 0.01). Based on students’ academic performance, the happiest group was students with good academic performance, whose mean is 24.22 while the standard deviation is 4.835. Followed by students with poor academic performance, whose mean is 25.0 and whose standard deviation is 2.646, and lastly, students with average academic performance, whose mean is 22.88, whose standard deviation is 4.514. According to these findings, academic achievement impacts happiness and knowledge of mental health resources, but the knowledge and beliefs about mental health remain constant.

**Table 7** shows the results of the MHL subscales and OHQ-SF scores by Students’ living situations. Results showed no significant difference (F 1.186; p 0.315) in the Knowledge-Oriented MHLS score of the student that lives with family (M 8.41; SD 1.79), dormitory (M 8.12; SD 1.931), friends (M 8.35; SD 1.309) and other (M 8.7; SD 1.62). This data demonstrates that mental health knowledge does not differ due to living situations. The scores of Belief-Oriented MHLS are not significantly different for their living situation (F= 0.819, p= 0.484). According to the results, students staying with their families were scoring a bit higher (M = 6.35, SD = 1.61) than those staying in the dormitories (M = 6.16, SD = 1.792), with friends (M = 5.95, SD = 1.669), and others (M = 6.05, SD = 2.124). The Resource-Oriented scores on the MHLS were also similar across the groups (F = 1.16, p = 0.325). The score ranged from 2.25 (SD=0.972) of dormitory residents to 2.5 (SD=0.688) of friends’ council residents. The same score message was for those living with family and other residents. Nonetheless, the OHQ-SF (happiness) score varied significantly between the different living situations (F=4.457, p=0.004). Students who lived in “other” arrangements reported the greatest happiness mean scores (M = 25.93, SD = 4.172) followed by those who lived with families (M = 23.47, SD = 4.564) and friends (M = 23.25, SD = 4.518) respectively. Dormitory residents reported the lowest happiness scores (M = 22.8, SD = 5.02). The study found that the arrangement of the space has an impact on happiness. In particular, dormitory living has been noted for contributing to lesser happiness.

**Table 7:** Comparison of MHLS and OHQ-SF scores by students’ living situation.

Variable	Living Situation	N	Mean (Mean)	SD	F	p
Knowledge-Oriented MHLS	Family	272	8.41	1.79	1.186	0.315
	Dormitory	107	8.12	1.931		
	With a Friend	20	8.35	1.309		
	Other	40	8.7	1.62		
Belief-Oriented MHLS	Family	271	6.35	1.61	0.819	0.484
	Dormitory	107	6.16	1.792		
	With a Friend	20	5.95	1.669		
	Other	40	6.05	2.124		
Resource-Oriented MHLS	Family	274	2.42	0.809	1.16	0.325
	Dormitory	107	2.25	0.972		
	With a Friend	20	2.5	0.688		
	Other	40	2.33	0.944		
OHQ-SF	Family	275	23.47	4.564	4.457	0.004
	Dormitory	107	22.8	5.023		
	With a Friend	20	23.25	4.518		
	Other	40	25.93	4.172		

**Table 8** depicts the comparison of mental health literacy (MHL) subscales and happiness (OHQ-SF) scores and their different family attitudes (Authoritarian, Democratic, Inconsistent, and Protective). No meaningful differences were found regarding the Knowledge-Oriented MHLS (F = 1.83, p = 0.141). Participants belonging to families with an inconsistent attitude had the highest average score (M = 9.0, SD = 1.541) followed by those with a protective attitude (M = 8.37, SD = 1.766). Thereafter came democratic attitudes (M = 8.35, SD = 1.569) followed by authoritarian attitudes (M = 8.16, SD = 2.128). The difference in Scores was Not Significant. The Belief-Oriented MHLS did not demonstrate significant differences (F=1.21, p=0.304). Participants from republican families had a mean score of 6.05. Participants from democratic families had a mean score of 6.48. Participants who grew up in a protective family had a mean score of 6.39 (SD = 1.516), and those in an authoritarian family had a mean score of 6.24 (SD = 1.743). The Resource-Oriented MHLS demonstrated a significant variance in family attitudes (F = 2.66, p = 0.048). Participants with authoritarian families had the highest mean comparison in parenting styles with a mean of 2.48 (SD = 0.776), followed by protective families with a mean of 2.39 (SD = 0.835), democratic families with a mean of 2.37 (SD =0.866) and inconsistent family with a mean of 2.0 (SD = 1.118). It can be interpreted from the findings that participants from the democratic family are fewer cellular phone addicts. This indicates that families’ attitudes probably have some impact on the awareness of mental health resources, with authoritarian families being more scored on probably impactful variable. There were significant differences for the OHQ-SF (Happiness Scores) (F = 9.89, p < 0.001). People from protective families were the happiest (M = 24.23, SD = 4.321). Moreover, those from democratic families were right behind them (M = 24.14, SD = 4.518). Participants coming from authoritarian families had significantly lower scores (M = 22.68, SD = 4.975). An inconsistent family-type participant was reported with the lowest happiness level (M = 20.06, SD = 4.632). The findings of the study also showed that family attitudes could play an important part in happiness, with greater family attitudes having better effects.

**Table 8:** Comparison of MHLS and OHQ-SF scores by students’ family attitudes.

Variable	Family Attitudes	N	Mean (Mean)	SD	F	p
Knowledge-Oriented MHLS	Authoritarian	104	8.16	2.128	1.83	0.141
	Democratic	131	8.35	1.569		
	Inconsistent	33	9.0	1.541		
	Protective	171	8.37	1.766		
Belief-Oriented MHLS	Authoritarian	104	6.24	1.743	1.21	0.304
	Democratic	130	6.05	1.871		
	Inconsistent	33	6.48	1.856		
	Protective	171	6.39	1.516		
Resource-Oriented MHLS	Authoritarian	104	2.48	0.776	2.66	0.048
	Democratic	133	2.37	0.866		
	Inconsistent	33	2.0	1.118		
	Protective	171	2.39	0.835		
OHQ-SF	Authoritarian	103	22.68	4.975	9.89	< .001
	Democratic	133	24.14	4.518		
	Inconsistent	34	20.06	4.632		
	Protective	172	24.23	4.321		

Lifestyle Factors and Their Influence on MHLS and OHQ-SF Scores are depicted in **Table 9**. The examination of lifestyle factors (smoking, alcohol use, exercise, and healthy nutrition) towards happiness (OHQ-SF) and mental health literacy (MHL) subscales presented several significant findings, especially for happiness and specific mental health literacy sub-scales.

**Table 9:** Lifestyle factors and their influence on MHLS and OHQ-SF scores.

MHLS and OHQ-SF	Variable	Response	N	Mean (Mean)	SD	F	p
Knowledge-Oriented MHLS	Smoking	Yes	112	8.37	1.884	2.79e-5	0.996
		No	326	8.37	1.768		
	Alcohol Use	Yes	107	8.36	1.627	0.00381	0.951
		No	332	8.37	1.847		
	Exercise Status	Yes	100	8.57	1.914	1.7	0.193
		No	339	8.3	1.756		
	Healthy Nutrition	Yes	231	8.37	1.962	0.00185	0.966
		No	208	8.36	1.591		
Belief-Oriented MHLS	Smoking	Yes	112	6.16	1.957	0.492	0.483
		No	325	6.29	1.619		
	Alcohol Use	Yes	107	6.47	1.574	2.08272	0.15
		No	331	6.19	1.747		
	Exercise Status	Yes	100	5.96	2.141	4.03	0.045
		No	338	6.35	1.551		
	Healthy Nutrition	Yes	230	6.23	1.727	0.14736	0.701
		No	208	6.29	1.693		
Resource-Oriented MHLS	Smoking	Yes	112	2.52	0.771	4.308	0.039
		No	328	2.32	0.884		
	Alcohol Use	Yes	108	2.44	0.846	0.72031	0.397
		No	333	2.35	0.865		
	Exercise Status	Yes	100	2.57	0.82	6.8	0.009
		No	341	2.32	0.864		
	Healthy Nutrition	Yes	233	2.42	0.847	1.18828	0.276
		No	208	2.33	0.873		
OHQ-SF	Smoking	Yes	112	22.18	5.349	12.558	< .001
		No	329	23.98	4.379		
	Alcohol Use	Yes	108	22.98	4.557	1.88364	0.171
		No	334	23.69	4.737		
	Exercise Status	Yes	101	24.77	5.444	9.47	0.002
		No	341	23.15	4.395		
	Healthy Nutrition	Yes	234	24.69	4.865	33.19192	< .001
		No	208	22.2	4.135		

### 4.1 Knowledge-Oriented MHLS

There were no significant differences among Knowledge-Oriented MHLS scores across all lifestyles. The means of smokers (M = 8.37, SD = 1.884) and the means of non-smokers (M = 8.37, SD = 1.768) were the same (F = 2.79e-5, p = 0.996). Smoking does not affect knowledge of mental illness. In the same way, drinkers (M = 8.36, SD = 1.627) and non-drinkers (M = 8.37, SD = 1.847) showed no difference in scores (F = 0.00381, p = 0.951). Participants who engaged in regular exercise (M = 8.57, SD = 1.914) scored higher than non-exercising participants (M = 8.3, SD = 1.756), but the difference was not significant (F = 1.7, p = 0.193). Again, no difference was observed with healthy nutrition habits since those who normally eat healthy (M = 8.37, SD = 1.962) and those who do not (M = 8.36, SD = 1.591; p = 0.966). The mental health literacy of young adults is not greatly affected by lifestyle choices.

### 4.2 Belief-Oriented MHLS

According to the results, the exercise status of participants was significantly associated with the Belief-Oriented MHLS score. Not exercising participants (M=6.35, SD=1.551) scored higher than exercising participants (M=5.96, SD=2.141), possibly owing to beliefs or attitudes imparted by lifestyle habits. For smoking (F = 0.492, p = 0.483), alcohol use (F = 2.08272, p = 0.15), and healthy nutrition (F = 0.14736, p = 0.701), there are no significant differences with comparable scores across groups. Thus, any differences in mental health beliefs seen among humans are not due to the aforementioned Lifestyle.

### 4.3 Resource-Oriented MHLS

There were notable differences in Resource-Oriented MHLS rates on smoking (F = 4.308, p = 0.039) and exercise status (F = 6.8, p = 0.009). Smokers (M = 2.52, SD = 0.771) were more familiar with the available mental health resources than non-smokers (M = 2.32, SD = 0.884). In the same way, subjects who train (M = 2.57, SD = 0.82) perform better than those who do not (M = 2.32, SD = 0.864). This finding implies that exercise may increase exposure to or knowledge of mental health resources. The study found that there were no significant differences in the use of alcohol (F = 0.72031, p = 0.397) and healthy nutrition (F = 1.18828, p = 0.276) based on the level of knowledge regarding mental health services.

### 4.4 OHQ-SF (Happiness Scores)

Smoking, exercise, and healthy nutrition significantly affected the happiness scores of people. Non-smokers (M = 23.98, SD = 4.379) were significantly happier than smokers (M = 22.18, SD = 5.349; F = 12.558, p < 0.001). Likewise, participants who exercised regularly (M = 24.77, SD = 5.444) were happier than those who did not exercise (M = 23.15, SD = 4.395; F = 9.47, p = 0.002). From the table, it can be observed that healthy nutrition (F = 33.19192, p < 0.001) registered the most significant difference since individuals who practice healthy eating (M = 24.69, SD = 4.865) reported much higher happiness than individuals who do not practice healthy eating (M = 22.2, SD = 4.135). The use of alcohol did not significantly affect the happiness levels (F = 1.88364, p = 0.171) of the participants who drank alcohol (M = 22.98, SD = 4.557) when compared with their counterparts who abstained from alcohol (M = 23.69, SD = 4.737).

According to the analysis in **Table 10**, the significance of health-related factors (perceived health status, chronic diseases) and mental health-related factors (psychological problems, family mental health) on the sub-scales of MHL and OHQ-SF happiness is examined.

**Table 10:** Comparison of MHLS and OHQ-SF scores by students’ health and mental health.

MHLS and OHQ-SF	Variable	Responses	N	Mean (M)	SD	F	p
Knowledge-Oriented MHLS	Perceived Health Status	Good	268	8.32	1.9	0.853	0.427
		Average	163	8.4	1.624		
		Bad	8	9.13	1.356		
	Chronic Illness	Yes	52	8.35	1.52	0.00389	0.95
		No	386	8.36	1.83		
	Chronic Illness Status in Families	Yes	211	8.53	1.741	3.51	0.062
		No	228	8.21	1.832		
	Psychological Issues	Yes	107	8.6	1.498	2.41	0.121
		No	332	8.29	1.875		
	Students’ Support seeking status	Yes	27	8.19	1.711	0.988	0.322
		No	122	8.54	1.677		
	Family Mental Health Status	Yes	48	8.58	1.442	0.802	0.371
		No	391	8.34	1.832		
Belief-Oriented MHLS	Perceived Health Status	Good	268	6.24	1.661	0.549	0.578
		Average	162	6.27	1.801		
		Bad	8	6.88	1.458		
	Chronic Illness	Yes	52	6.5	1.407	1.17612	0.279
		No	385	6.23	1.747		
	Chronic Illness Status in Families	Yes	211	6.38	1.638	1.97	0.161
		No	227	6.15	1.769		
	Psychological Issues	Yes	107	6.26	1.55	9.59e-05	0.992
		No	331	6.26	1.76		
	Students’ Support seeking status	Yes	27	6.63	0.839	2.716	0.101
		No	122	6.0	1.941		
	Family Mental Health Status	Yes	48	6.58	1.182	1.93	0.165
		No	390	6.22	1.76		
Resource-Oriented MHLS	Perceived Health Status	Good	270	2.39	0.858	0.159	0.853
		Average	163	2.36	0.873		
		Bad	8	2.25	0.707		
	Chronic Illness	Yes	52	2.31	0.853	0.41761	0.518
		No	388	2.39	0.854		
	Chronic Illness Status in Families	Yes	212	2.42	0.82	1.4	0.237
		No	229	2.33	0.895		
	Psychological Issues	Yes	107	2.27	0.875	2.04	0.154
		No	334	2.41	0.854		
	Students’ Support seeking status	Yes	27	2.41	0.694	0.677	0.412
		No	122	2.25	0.965		
	Family Mental Health Status	Yes	49	2.31	0.871	0.345	0.558
		No	392	2.38	0.859		
OHQ-SF	Perceived Health Status	Good	271	24.6	4.591	23.323	< .001
		Average	163	22.0	4.2		
		Bad	8	18.0	6.0		
	Chronic Illness	Yes	52	21.85	4.94	7.69023	0.006
		No	389	23.76	4.626		
	Chronic Illness Status in Families	Yes	213	22.99	4.659	5.37	0.021
		No	229	24.02	4.691		
	Psychological Issues	Yes No	108	21.8	4.581	20.07	< .001
			334	24.08	4.606		
	Students’ Support seeking status	Yes	28	23.0	4.546	1.21	0.273
		No	122	21.95	4.552		
	Family Mental Health Status	Yes	49	21.41	4.873	11.399	< .001
		No	393	23.78	4.615		

### 4.5 Knowledge-Oriented MHLS

Most health-related factors didn’t show any significant change in Knowledge-Oriented MHLS score. Perceived health status did not have a significant effect on knowledge scores (F = 0.853, p = 0.427). Good health status students (M = 8.32, SD = 1.9); average health status students (M = 8.4, SD = 1.624); bad health status students (M = 9.13, SD = 1.356). Likewise, the presence of chronic illness (F = 0.00389, p = 0.95) or family chronic illness history (F = 3.51, p = 0.062) demonstrated no significant differences. Students with psychological issues had slightly more knowledge (M = 8.6, SD = 1.498) than students with no psychological issues (F = 2.41, p = 0.121). However, it was not statistically significant. The knowledge-oriented literacy was established to have no significant results for the utilization of services by the families. Also, the families’ mental health was seen not to have significant results as well as the families’ tendency to seek out support for help.

### 4.6 Belief-Oriented MHLS

There are no meaningful differences in the Belief-Oriented MHLS scores on perceived health status (F = 0.549, p = 0.578), chronic illness (F = 1.17612, p = 0.279) or family chronic illness status (F = 1.97, p = 0.161). Psychological problems did have a significant impact (F = 9.59e-05, p = 0.992) as the scores of students with psychological problems (M = 6.26, SD = 1.55) and without psychological problems (M = 6.26, SD = 1.76) were similar. In terms of support-seeking behavior, students who sought support scored higher (M = 6.63, SD = 0.839) compared to students who did not seek support (M = 6.0, SD = 1.941) but it was nearly significant (F = 2.716, p = 0.101) Family mental health was not statistically significant (F = 1.93, p = 0.165).

### 4.7 Resource-Oriented MHLS

The MHLS scores that are resource-oriented were not affected by health status conditions or chronic disease. (F value = 0.159, P value = 0.853;F value = 0.41761, P value = 0.518). A survey of students with occupational therapy shows no significant difference in their chronic illness between the two groups of students (F = 1.4, p = 0.237). It was because students with and without family histories of chronic illness show no significant difference. The same goes for students with and without psychological problems. In the same way, support-seeking behavior (F = 0.677, p = 0.412) and mental health issues in the family (F = 0.345, p = 0.558) had no significant effect. These findings suggest that knowledge of and access to mental health resources are stable across these health variables.

### 4.8 OHQ-SF (Happiness Scores)

Multiple health-related factors significantly impacted happiness levels (OHQ-SF). The effect of perceived health status was significant. (F=23.323, p<0.001). Students who reported good health have the highest scores (M=24.6, SD=4.591). Followed by those who reported with average health (M=22.0, SD=4.2) and bad health (M=18.0, SD=6.0). Happiness was significantly influenced by chronic illness (F = 7.69023, p = 0.006), where students without chronic illness (M = 23.76, SD = 4.626) scored higher than those with chronic illness (M = 21.85, SD = 4.94). The family’s chronic illness status illustrates significant effects, F = 5.37, p = 0.021. Nonetheless, students coming from families with no history of chronic illness demonstrated higher mean scores, M = 24.02, SD = 4.691, than those with a history of family chronic illness, M = 22.99, SD = 4.659. Psychological issues also played an essential role (F=20.07, p<0.001). The non-psychological issues group showed higher marks (M=24.08, SD=4.606) in comparison to the psychological issues group (M=21.8, SD=4.581). In conclusion, family mental health issues have significantly impacted happiness (F=11.399, p<0.001). The students from families without any mental health issues had a higher score (M=23.78, SD=4.615) compared to those with family mental health issues (M=21.41, SD=4.873) in the scale measuring happiness.

**Table 11** performs the analysis regarding the correlations among the students’ happiness outcomes as measured by the OHQ-SF and the three subscales of mental health literacy Knowledge-Oriented, Belief-Oriented and Resource-Oriented MHLS. The findings show that relationships between the variables of interest are both significant and non-significant.

**Table 11:** Correlation between students’ happiness levels and mental health literacy.

Variable	Pearson's r	p-value
Knowledge-Oriented MHLS - Belief-Oriented MHLS	-0.119 *	0.013
Knowledge-Oriented MHLS - Resource-Oriented MHLS	0.117 *	0.014
Knowledge-Oriented MHLS – OHQ-SF	-0.036	0.450
Belief-Oriented MHLS - Resource-Oriented MHLS	-0.111 *	0.020
Belief-Oriented MHLS – OHQ-SF	-0.056	0.247
Resource-Oriented MHLS – OHQ-SF	0.117 *	0.014

*p* < .05 (*) indicating statistical significance at the 0.05 level.

The findings revealed a significant negative correlation (r = -0.119, p = 0.013) between the Knowledge-Oriented MHLS and Belief-Oriented MHLS sub-scales, suggesting that individuals with a higher level of mental health knowledge possess a slightly lower level of belief and attitude towards mental health. This finding could mean that people with more knowledge are less influenced by subjective beliefs. That is, people with a lot of factual knowledge may not be that influenced by subjective knowledge/beliefs.

A positive and significant correlation (r = 0.117, p = 0.014) was noted between the Knowledge-oriented MHLS and Resource-oriented MHLS, indicating that students who possess more knowledge about mental health are also aware of the resources available to them. This connection shows that knowledge could help someone get or know about resources, and it’s an essential part of mental health literacy. On the negative side, there is a significant correlation between the Resource-Oriented MHLS and the Belief-Oriented MHLS (r = −0.111, p = 0.020). That means that with the increase in beliefs and attitudes, the familiarity will decrease and vice-versa. The finding may show that people feel they can rely on beliefs if they do not have direct experience with tangible resources, possibly due to different exposure to mental health or education.

Since the correlation between Resource-Oriented MHLS and happiness score was 0.117 and significant at 0.05 level, it can be stated that the students who are familiar with mental health resources have higher happiness scores. The findings suggest that being aware of and having access to resources can lead to feelings of calmness. Furthermore, being able to access resources could provide the support needed when one is feeling stressed. Nevertheless, the study did not find a statistically significant correlation between the Knowledge-Oriented MHLS and happiness scores (r = −0.036, p = 0.450). As a result, this shows that they have an inverse relationship. The Mental Helplessness Scale (MHLS) score regarding a person’s belief was related to the score on the happiness scale. The correlation was also insignificant. Also, the correlation was negative in value (-0.056), and the p-value was also significant (0.247).

The results reveal that some aspects of mental health literacy, like knowing where help is available, are very closely related to happiness. However, knowing and their beliefs are not so strongly related to happiness. The inverse correlations between subscales such as Knowledge-Oriented and Belief-Oriented MHLS or Belief-Oriented and Resource-Oriented MHLS indicate that students may reasonably be integrating the two. For example, students who emphasize facts may de-emphasize beliefs, and students who believe in something may not care much about things. An intervention to enhance mental health literacy may improve the mental well-being of the public, and encourage an effective help-seeking process.

These outcomes reveal that mental health literacy and happiness have a complex relationship. Using practical resources may be positively related to emotional well-being, whereas wider knowledge or belief-driven factors may have an indirect relationship or require fuller investigation of their interactions. The analysis shows targeted intervention areas that need to be looked at, especially in relation to access to resources and integration of knowledge with beliefs to promote mental health literacy and happiness among students.

**Table 12** presents the descriptive statistics of scores obtained by students for the subscales of the Mental Health Literacy Scale (MHLS) namely Knowledge-Oriented, Belief-Oriented, Resource-Oriented, Oxford Happiness Questionnaire (OHQ-SF) and Total MHLS. This information shows what was most likely to occur, the distance of scores from one another, and how far away the scores were. The mean for the Knowledge-Oriented MHLS subscale was 8.36 (SD = 1.79) with a median of 9.0 with most students scoring at the higher end. The minimum score was 0.0, and the maximum score was 10.0, which reflects a huge variation in the knowledge of participants. The accumulated score on this subscale was 3672.0 which means the students had a good level of knowledge about the same. The average score on the Belief-Oriented MHLS subscale (score of 6.26) was higher than for the Knowledge-Oriented subscale. Its range (i.e., lowest to highest score) was a little narrower, however (1.71), compared with the other subscale. The median on this subscale also indicates that the higher score (i.e., 7.0) was more common than the lower scores. The range of score is from 0.0 to 8.0 and the total sum is 2742.0. Based on these results, it can be concluded that students’ health beliefs and attitudes are fairly varied; although most are weighted towards the higher end. For this Resource-Oriented MHLS subscale, the mean was 2.37 (SD = .86), and the median was 3.0. Scores with a range of 0.0 as minimum and 4.0 as maximum had a narrow range. The whole score was 1047.0 which means low scores overall in all other subscales. This implies that even though students have good knowledge and beliefs regarding mental health, they have little knowledge about the resources.

**Table 12:** The scores that students obtained from the scales and subgroups.

Descriptive	Knowledge-Oriented MHLS	Belief-Oriented MHLS	Resource-Oriented MHLS	OHQ-SF	MHLS Total
N	439.0	438.0	441.0	442.0	436.0
Missing	4.0	5.0	2.0	1.0	7.0
Mean	8.36	6.26	2.37	23.5	17.0
Median	9.0	7.0	3.0	23.0	17.0
Sum	3672.0	2742.0	1047.0	10396.0	7414.0
Standard deviation	1.79	1.71	0.86	4.7	2.48
Minimum	0.0	0.0	0.0	9.0	7.0
Maximum	10.0	8.0	4.0	35.0	21.0

The mean for the OHQ-SF (Happiness) was 23.5 (SD=4.7), the median was 23.0. The minimum score was 9.0 and the maximum score was 35.0, reflecting considerable variation in the students’ happiness. The overall average happiness is moderate, summarizing a total score of 10396.0. Ultimately, the average for the MHLS Total score, which is the value of the three subscales, was 17.0 (SD = 2.48) with a median of 17.0, minimum of 7.0, and maximum of 21.0. Taking all the dimensions together, the sum was 7414.0, indicating a moderately high mental health literacy level. These stats show key patterns in students’ mental health literacy and happiness. Information and beliefs around mental health are relatively strong, but knowledge of resources is weak. Moreover, the high variation in the happiness score makes one further probe who is responsible for their happiness. With mostly equal median and mean values across most scales, the distributions can be said to be fairly symmetric. This report can be a basis for understanding the relationship between mental health literacy and happiness.

## 5 Discussion

The relationship between mental health literacy (MHL) and happiness among university students was analyzed broadly. Further, the acceptance of the influence of demographic, lifestyle, health, and psychological factors was discussed. The study results fit well with established theories like Keyes’ Dual-Continua Model and improve on what is already known while offering fresh insights.

MHL shows notable differences based on gender, age, and demographic findings. Women demonstrated greater Knowledge-Oriented MHL, probably due to norms and expectations around emotions, help-seeking, etc. Some studies have shown that knowing more does not always make one happier. Being informed may not improve one’s feelings or emotions (27). According to Pedrelli et al., women are likely to experience a depression gap due to the demands of multitasking and stress, which negates the power of knowledge. On the other hand, men had lower Knowledge-Oriented scores for MHL, which may reflect how stigma and masculine ideologies still discourage emotionality (22). The small gender differences in MHL signal a universal need for improvement among all students.

Students aged 21–25 had the highest score on Knowledge-Oriented MHL. This is possibly due to their greater exposure to academic and social pressures and increased opportunities to use such mental health resources. Nevertheless, the lack of significant differences in happiness across age groups suggests that family support, resilience, and access to resources may be more important in promoting happiness (36).

Family dynamics became an essential determinant of happiness. Students from democratic and protective family setups feel happier than other children. Families with authoritarian or inconsistent dynamics were correlated with lower happiness but slightly higher Resource-Oriented MHL levels. This could show that people lean on outside help because family members are missing (39). The study’s results enhance the significance of family-based psychoeducation, proving it to be an important addition to any mental health intervention.

Lifestyle and health factors also played a critical role in happiness and MHL. According to Vaillant, it is a well-established fact that those with better physical health levels tend to be happier, as it is thought that physical and mental health are interconnected. Exercise regularly and eating healthily are strongly related to higher Resource-Oriented Mental Health Literacy (MHL) and happiness. This finding is consistent with existing literature showing that the relationship between physical and mental health is bidirectional (24). On the other hand, smokers had a greater Resource-Oriented MHL than non-smokers but were less happy. Thus, knowing about resources does not help.

Happiness also depended on living arrangements. College students who lived in dorms reported lower happiness scores than at home or with friends. This result shows that social support can help to relieve stress and improve emotional stability. Dormitories lacking stable support systems may add to academic pressure and social isolation (40). Initiatives focused on community-building in dorms may fill this gap and enhance students’ well-being.

Academic success was strongly associated with happiness, with students who performed better reporting more significant happiness levels. This indicates that children experiencing academic success tend to be emotionally stable and resilient. When students do well, they feel more purposeful, accomplished, and confident, contributing to happiness (11). On the other hand, poor academic achievement may lead to increased stress and anxiety in competitive universities. The significance of bridging academic counseling with mental health support is highlighted in the findings as students’ mental health and academic performance impact shaping in school.

The correlation analysis provided important information about the relationship between mental health literacy (MHL) subscales and happiness.

### 5.1 Resource-Oriented MHL and Happiness

The greatest positive connection was noticed in Resource-Oriented MHL with happiness. This shows that people will be happy if they have access to knowledge and resources. Students with high resource-oriented MHL were most likely to be confident in seeking help when things got difficult, thus reducing stress and promoting resilience. According to Keyes (36), the Dual-Continua model supports the notion that resource utilization plays a promotive function for mental health. Moreover, this outcome lends credence to Fredrickson’s (37) broaden-and-build theory, which describes how positive emotions– emotions elicited through resource accessibility– broaden our thoughts and actions and foster happiness and resilience (37). The strong connection between these two variables highlights the importance of interventions enhancing student awareness and resource access.

### 5.2 Knowledge-Oriented MHL and Happiness

The link between Knowledge-Oriented MHL and happiness was less strong, showing that knowledge does not always lead to happiness. Students with higher knowledge scores may have greater recognition of mental health problems, but this awareness does not make them feel any less distressed or more flourishing. Previous studies have found that knowledge-based interventions seldom target the emotional and behavioral aspects essential for well-being (26). Knowing all the reasons that should make us happy is different from actually doing it. This means that theories should not try to stick to the ‘ideal happy state’ but rather focus on skills to help us manage our day-to-day emotional experiences.

### 5.3 Belief-Oriented MHL and Happiness

The finding showed that Belief-Oriented MHL and happiness had an insignificant or weak relationship, indicating that subjective mental health attitudes may not directly influence emotional feelings. According to Griffiths et al. (22), this finding is consistent with criticisms of belief-based approaches, which emphasize individual attitudes but overlook systemic or environmental structures that facilitate well-being. Some correlations were negative, specifically in the case of Knowledge-Oriented MHL and Belief-Oriented MHL, which might suggest a contradiction between knowledge and beliefs. Conflicting mental models, such as stigmatized attitudes versus evidence-based awareness, may prevent students from acting knowledgeably on their symptoms, which is reflected in this disparity. Initiatives to reduce stigma may help to reconcile these dimensions.

The interplay between the MHL subscales provided added insight. As an illustration, the Resource-Oriented MHL positively correlates with both the Knowledge-Oriented and Belief-Oriented MHL, meaning that resource awareness is usually built on knowledge and beliefs. The weak relationship between knowledge-oriented and belief-oriented mental health literacy (MHL) calls for integrative interventions to align them. The correlation patterns suggest that the MHL dimensions have different levels of importance for promoting happiness. Resource-Oriented MHL is probably the construct influencing well-being enhancement through the most directly actionable pathways. In contrast, Knowledge-Oriented MHL and Belief-Oriented MHL are arguably too far removed from practical outcomes to be strong enough. These ideas highlight the importance of utilizing resources when building mental health literacy knowledge and belief systems in a multi-faceted manner. The descriptive statistics accentuate the gap between MHL and students’ happiness in which a significant variation exists in the Resource-Oriented MHL score. This variation emphasizes the need for targeted interventions that cater to specific subgroups’ needs.

To sum up, the study shows a very complex relationship between MHL and Happiness. It emphasizes the need for resource-oriented interventions, gender sensitivity, and enabling environments for emotional well-being. According to these results, mental health literacy significantly contributes to happiness. It provides further insight and guidance for future studies and practices.

## 6 Theoretical and practical implications

### 6.1 Theoretical implications

The research study’s results coincide with the work of Keyes (36) in the Dual-Continua Model, which argues that mental health is more the opposite of the absence of mental illness but rather the joint occurrence of positive mental states and the absence of psychological distress. The strong relationship between Resource-Oriented MHL scores and happiness shows that awareness of mental health resources plays a crucial role in happiness. This gives weight to the idea that flourishing can be promoted through both preventing and promoting practices, as emphasized in Keyes’ model (38); the findings indicating no other significant correlations between MHL subscales such as Knowledge-Oriented and happiness imply that knowledge may not be enough to help someone flourish. In other words, research should focus on self-efficacy, social support, and coping strategies that mediate the effect of mental health literacy on mental well-being.

Fredrickson (37) developed a useful broaden-and-build theory of positive emotions in interpreting this finding. Fredrickson’s broaden-and-build theory states that positive emotions widen one’s thoughts-and-action repertoire. Moreover, it builds lasting personal resources. According to the study, access to mental health resources, whether real or imagined, triggers positive emotions and reinforces happiness. For instance, students aware of the available resources tend to experience less stress as they know they can find help when faced with difficulties. Research into whether these factors happen simultaneously or whether one brings about the other is called causation.

The research also provides insight into gender dynamics and mental health literacy. Women scored higher than men on knowledge-oriented mental health literacy, which means they are better at acquiring mental health knowledge and applying it to their lives because they do the work on themselves emotionally more than men do. The finding suggests that women’s greater engagement with emotions may build resilience from using resources. This is according to Fredrickson’s theory. However, gender-based interventions are needed to tackle coping strategies, including enhanced stress and anxiety among females (27).

The study contributes to understanding family dynamics and their link to mental health and happiness. The positive implications of democratic and protective family orientations are connected to Keyes’ focus on the social dimension of flourishing, whereby relationships and social environments are important (39). These findings highlight the importance of studying the relationship between family support and resource-oriented literacy and emotional health to design holistic intervention models.

### 6.2 Practical implications

The result gives rise to several recommendations to universities, policymakers, and mental health professionals to enhance students’ mental health literacy.

1. Designing deliberate mental health literacy programs: Universities must develop holistic MHL programs that maintain knowledge and promote using resources and practically getting help. These programs could include digital and in-person workshops on identifying and navigating campus mental health services. Role-playing exercises are simulations that help reduce stigma and encourage students to seek help. MHL sessions should be made compulsory during student orientation or as part of the general education curriculum to reach all students early (26).
2. Promoting gender-sensitive strategies: Tailored interventions are needed, given the gender differences in mental health literacy. The campaigns for male students must address the cultural norms and stigma that stop them from seeking help. It can include peer mentors or male role models to engage them. Programs must promote the ability to use mindfulness, coping skills, and other stress-reducing strategies (40).
3. Making academic course of education mental health: Mental health literacy should become part of the academic curriculum. Courses on MHL could be included in the core syllabus of fields like psychology, sociology, and health sciences. Students not majoring in health-related fields could take elective courses on well-being, which could help normalize mental health and happiness (24).
4. Improving access and visibility of resources. Low Resource-Oriented MHL scores show gaps in resource awareness—ways to beam up the profile.
  - Digital Platforms: Affordable and easy-to-use mobile apps or websites providing central access to campus mental health information. Campus campaigns will be held regularly to create awareness of the available counseling services and de-stigmatization.
  - Having resource guides in dorms, libraries, and student centers is a good idea since that is where most students go (22).
5. Incorporating family-centered interventions: It shows us that family-based programs are necessary. Families could engage through universities.
  - Workshops for Parents: Teaching democratic and supportive parenting styles. We hold sessions with families to communicate openly with students.
  - Family Counseling Programs: Support students struggling with family relationships (39).
6. Zoning in on subsets of at-risk behavior: Insights obtained from smokers and unhealthy lifestyles are a call for action. Campaigns targeting smokers can use their knowledge of healthcare to promote a wider array of services. Customized programs for sedentary or unhealthy eaters could also incorporate physical and mental health literacy (25).
7. Using technology for MHL interventions: Digital platforms have solutions that can grow with the need for MHL. Mobile applications and digital subscriptions could be afforded.
  - Self-Paced Learning: Mental health literacy and coping strategy modules.
  - Availability of Counseling and Peer Support in Real Time.
  - Anonymous assistance refers to online help students can access if they are unwilling to seek face-to-face assistance (41).
8. Creating institutional guidelines on mental health: Policymakers must incorporate mental health into institutional strategic planning. This entails financing for counseling centers, routine evaluations of campus requirements, and compulsory MHL training for all new students (20).
9. Evaluating program effectiveness: We should study all interventions to know their level of effectiveness. Strategies for developing the program reaches will be provided together with the revised logic model in Section 5.

## 7 Future research directions

Future studies should adopt longitudinal designs to investigate how mental health literacy (MHL) changes happiness. Such work will help to pinpoint causality and the longer-term impact of intervention. Researching these varied concepts in different cultures, institutions, and family situations would give more insight into their roles and impact on MHL and happiness. Similarly, the availability of resources matters too. Also, research into gender-specific interventions is important. One should look into reducing stigma for male populations. Moreover, one should also research stress and resilience-building interventions for women. Including self-efficacy, coping strategies, and emotional intelligence into the mental health literacy (MHL) frameworks would enhance understanding of how MHL can lead to positive outcomes. Researching the behavioral outcomes of MHL related to help-seeking and lifestyle modification could make awareness beneficial. Tech solutions like mobile applications and online counseling tools must also be evaluated to improve MHL and emotional well-being, especially among under-resourced people. In summary, future research can examine how academic performance may lead to the development of stress, which ultimately results in happiness. These directions provide roads for progressing with mental health literacy and happiness and related research, policy, and practice.

## 8 Limitations of the study

Limitations of this study include its reliance on self-reported data, which may introduce bias due to social desirability or inaccurate self-assessment. The sample was drawn from a single private university in Istanbul, limiting the generalizability of the findings to other institutions or cultural contexts. The study used a cross-sectional design, preventing causal inferences between mental health literacy and happiness. Additionally, the study focused on quantitative data and did not explore deeper psychological or contextual factors affecting these variables. Finally, potential confounding factors, such as personality traits and life experiences, were not considered, which may influence the observed relationships.

## 9 Conclusion

The research outcomes shed light on the relationship between mental health literacy (MHL) and the happiness of university students with reference to their demographic, health, lifestyle, and psychological characteristics. The results point to the importance of Resource Oriented MHL in improving emotional well-being, as happiness increases when we are aware of and have access to mental health resources. Weaker correlations between Knowledge-Oriented and Belief-oriented mental Health Literacy (MHL) and happiness show that knowledge and beliefs are insufficient without actions and support. Research has shown that there are differences in MHL between genders. For example, while women (on average) trump men in MHL studies, they report lower happiness than men. The health and lifestyle behaviors of a person’s family further dictate the outcome of mental health problems. The differences in MHL and happiness by subgroup call for equity interventions focusing on the sub-groups contexts like academic pressure and dormitory isolation. This research highlights the need for resource-oriented literacy in mental health, gender-specific approaches, and an enabling environment. By tackling these multifarious needs of students, universities, policymaking bodies, and mental healthcare officials can build resilience, alleviate distress, and promote flourishing among students. This understanding of MHL contributes to happiness and is a primary foundation for future research and applications.

## Data Availability

The raw data supporting the conclusions of this article will be made available by the authors, without undue reservation.
